# Super-ranging. A new ranging strategy in European badgers

**DOI:** 10.1371/journal.pone.0191818

**Published:** 2018-02-14

**Authors:** Aoibheann Gaughran, David J. Kelly, Teresa MacWhite, Enda Mullen, Peter Maher, Margaret Good, Nicola M. Marples

**Affiliations:** 1 Department of Zoology, School of Natural Sciences, Trinity College, Dublin, Ireland; 2 Department of Agriculture, Food and the Marine, Kildare Street, Dublin, Ireland; 3 National Parks and Wildlife Service, Department of Culture, Heritage and the Gaeltacht, Wicklow Mountains National Park, Kilafin, Laragh, Wicklow, Ireland; Sichuan University, CHINA

## Abstract

We monitored the ranging of a wild European badger (*Meles meles)* population over 7 years using GPS tracking collars. Badger range sizes varied seasonally and reached their maximum in June, July and August. We analysed the summer ranging behaviour, using 83 home range estimates from 48 individuals over 6974 collar-nights. We found that while most adult badgers (males and females) remained within their own traditional social group boundaries, several male badgers (on average 22%) regularly ranged beyond these traditional boundaries. These adult males frequently ranged throughout two (or more) social group’s traditional territories and had extremely large home ranges. We therefore refer to them as super-rangers. While ranging across traditional boundaries has been recorded over short periods of time for extraterritorial mating and foraging forays, or for pre-dispersal exploration, the animals in this study maintained their super-ranges from 2 to 36 months. This study represents the first time such long-term extra-territorial ranging has been described for European badgers. Holding a super-range may confer an advantage in access to breeding females, but could also affect local interaction networks. In Ireland & the UK, badgers act as a wildlife reservoir for bovine tuberculosis (TB). Super-ranging may facilitate the spread of disease by increasing both direct interactions between conspecifics, particularly across social groups, and indirect interactions with cattle in their shared environment. Understanding super-ranging behaviour may both improve our understanding of tuberculosis epidemiology and inform future control strategies.

## Introduction

Knowledge of ranging behaviour is particularly important where infectious diseases are problematic [1]. European badgers (*Meles meles)* are highly susceptible to tuberculosis (TB), caused by *Mycobacterium bovis* [2]. In continental Europe, badgers infected with TB have been reported in both France and Spain [3], with badgers becoming the focus of epidemiological studies in France [4,5]. In both the UK and Ireland badgers have been implicated in the spread of *M*. *bovis* to cattle and in acting as a wildlife reservoir for bovine tuberculosis [6–8]. The UK and Irish governments spend millions each year attempting to eradicate bovine tuberculosis (bTB), in part through culling badgers [7,9]. However, badgers are a protected species in both countries and culling is not considered a sustainable bTB control strategy [10]. Both the UK and Ireland are investigating parenteral and oral vaccine strategies designed to inoculate badgers against *M*. *bovis* [11–13]. However, to understand the dynamics of a disease and to control it successfully, a complete picture of the ecology and ranging behaviour of the carrier species is required [1]. The ranging of badgers is of direct importance to the transmission of TB infection both between individual badgers [14,15] and between badgers and cattle [16–20].

Badgers display flexibility in their social organisation, from mated pairs with offspring occupying large home ranges which may not be contiguous (*e*.*g*. [21,22] to large social groups defending small, stable, contiguous territories (*e*.*g*. [23,24]. This flexibility appears related to local population density and may be driven by factors such as variation in temperature or food resources [25–27] or artificial depression below carrying capacity due, for example, to persecution or culling [28–31]. Similarly, badgers display variation in ranging behaviour *e*.*g*. in environments where territoriality is reduced badgers do not mark boundaries between social groups [32–34] and in response to reductions in population density they may make increased extra-group excursions [29,30] or travel greater distances [31]. The evidence for sex-specific differences in ranging behaviour is equivocal. Some studies report no differences in movement patterns between male and female badgers [27,35–38]. Other studies report sex-specific differences in ranging, but do not agree whether males or females range further [15,21,22,24,39–42].

The current badger study was initiated by the Irish Department of Agriculture, Food and The Marine (DAFM) and National Parks and Wildlife Service (NPWS) [43]. It was designed to examine the effects of a major road upgrade and realignment project on the ranging behaviour of the local badger population using GPS tracking collars. This study was one of the longest ongoing GPS studies of its kind for this species (April 2010- August 2016). GPS tracking provided a much higher resolution view of behaviour than is practical using radio-tracking [44]. It generated a very large dataset (82 individual badgers yielding 103,001 GPS locations over 26,522 collar-nights) allowing us to take continuous readings of the same individual over months and in some cases over several years (mean 323 badger-nights per badger, range 12–1166). In our study area, the badgers showed seasonal variation in ranging behaviour, with home ranges reaching their maximum size in Summer (S1 Table). Here, we seek to reveal the extent to which there is a sex difference in ranging behaviour and the extent to which badgers habitually range beyond the boundaries of their social group territory. This data will contribute to the improved modelling of disease dynamics and the implementation of successful TB control strategies.

## Methods

### Study area

The study was conducted in Co. Wicklow, Ireland (52.924130, -6.117960). The study area was a matrix of undulating agricultural land (75%), with patches of mixed and coniferous woodland (14%) with small residential areas and farmyards scattered throughout (7%). Local farming practices include pasture (cattle and sheep), arable (primarily wheat, barley and maize) and some equestrian activity. In any given year, on average 68.5% of agricultural land was under pasture, while at least 16.7% was arable crops. A well-developed hedgerow system connected fields. The road upgrade involved building a new 16km section of motorway (M11) in which wildlife underpasses were installed, alongside the original national road (N11) [43]. The study area has expanded incrementally over the years from 18.5km^2^ in 2010 to 32.7km^2^ in 2016. The size of the study area in each year was estimated in ArcMap™ (ArcGIS® 10.4) by drawing a polygon around the GPS locations for that calendar year.

### Trapping and handling of badgers

Ethical approval for the project was granted by Trinity College Dublin’s Animal Research Ethics Committee (Project No. 290516) and the Health Products Regulatory Authority (Project No. 7024754). Badgers were captured under licences (NPWS Nos. 101/2009, 04/2010, 13/2010, C123/2010, 03/2011, C040/2011, C03/2013, C005/2013 and C001/2015) as required by the Wildlife Act, 1976, in two trapping events per annum: April-May (3–4 weeks) and September-October (3–4 weeks) using cage traps (following the methods in [45]) and, on occasions when necessary, stopped body restraints with a minimum closure of 32.5cm [46]. Cage traps were of a standard DAFM approved design, 1.1 metre to 1.3m long, about 35 cm wide and 35 cm high, and were constructed from 3cm square 8 gauge galvanised mesh, hot dipped and finished in a smooth black plastic coating (Rathcormac Steel Supplies, Rathcormac, Co. Sligo, Ireland). The triggering mechanism was by means of a string trip-line, the breaking of which closed the trap door behind the badger. Both cage traps and stopped restraints conformed to national legislation for humane trapping defined in the Wildlife Act, 1976, Regulations 2003 (S.I. 620 of 2003). Stopped restraints were used at setts where no badgers had been caught in cages and where badger activity was evident indicating the presence of “cage-shy” badgers (cages 97%, stopped restraints 3% of captures). Cages were baited with peanuts for two weeks prior to and during the trapping event. All fieldwork was carried out with the land owners’ consent. Data collection began in April 2010 in advance of roadworks commencing in September 2013. The roadworks were completed in August 2015, and GPS tracking continued until October 2016.

Captured badgers were anaesthetised in-cage by veterinary practitioners from DAFM using ketamine hydrochloride (Narketan 10® or Vetalar®) at 10 mg/kg and medetomidine (Domitor® or Medetor®) at 0.1 mg/kg [47]. This dose was delivered by intramuscular (i/m) injection into the lumbar muscles using a pole syringe. All badgers were marked by an implanted Radio Frequency Identification (RFID) microchip and a tattoo on first capture. The last 4 digits of the microchip number were tattooed to the right medial thigh (inside hindleg). The tattoo and microchip numbers were used to identify individual animals at subsequent recaptures during the study. Badgers were vaccinated against TB with Bacille Calmette-Guérin (BCG) vaccine by i/m injection into the lumbar muscles. All badgers were weighed, clinically examined for signs of ill-health, external wounds and parasite load, and records taken. Blood samples and pharyngeal swabs were taken to determine TB infection status (BrockTB Stat-Pak ® and selective mycobacterial culture respectively).

### Ageing and sexing of badgers

Age was determined by dentition [48,49] and general appearance of each badger. Cubs were defined as badgers in their first year, yearlings as badgers in their second year and adults as badgers in their third year or over. Badgers were assigned to a social group based on their most frequent trapping location and the GPS tracking data.

### Data collection

We aimed to capture as many badgers as possible within each social group. Badgers weighing 8kg or more and with a suitable neck to head ratio (that is, a cranial circumference of at least 1cm more than the neck circumference) were fitted with a Tellus Light (previously Tellus 1C) GPS collar (Followit Wildlife, Lindesberg, Sweden). This meant that collars weighed no more than 3% of a badger’s body weight. Such criteria usually precluded the collaring of cubs, except perhaps during autumn trapping events when they were at least 9 months old, and heavy enough to wear a collar. If badgers were recaptured in the same trapping session, they were identified, recorded and released without anaesthetic. Badgers wearing collars from a previous session had their collars replaced to ensure sufficient battery life for the new season. Collars were programmed to take four GPS readings per night at 10pm, 11pm, 1am and 2am, when badgers were expected to be above ground, except in April, May and September when 8 readings a night where taken hourly between 9pm and 4am [43].

### Data analysis

Over the course of the study 139 badgers were trapped and 82 of those were collared. As home ranges more accurately reflect physical/geographic borders between social group territories in Summer when home ranges are at their maximum size [50, 51, S1 Table], the data were subsetted for June, July and August months. GPS data from each collared individual for June, July and August in a given year were combined to estimate individual summer home ranges for that year. Home ranges were estimated using 95% Minimum Convex Polygons [52] calculated using the package “adehabitatHR” [53] in R Version 3.3.1. [54]. MCP areas were calculated and MCP shapefiles were generated and plotted in ArcMap. Plotting the data revealed an unusual ranging behaviour among some adult males. Their home ranges crossed “traditional” social group boundaries that other members of their respective social group rarely crossed. Further, this ranging behaviour was sustained for longer than 6 weeks *i*.*e*. apparent home range size was not merely an artefact of opportunistic mating, foraging or pre-dispersal exploratory forays into neighbouring ranges. Neither were these males in the process of dispersing as they did not permanently move away from their natal group. Instead, they were engaging in habitual extended ranging behaviour *i*.*e*. ranging beyond the traditional boundary of their social group, and using this area to the same extent as the area of their traditional home range. Hereafter, we refer to these adult males as “super-rangers” (SRs) and to adult males that did not maintain super-ranges as “traditional rangers” (TRs).

In order to investigate the unusual ranging behaviour observed in SRs, the dataset was further filtered. Cubs and yearlings were excluded as badger home range size naturally increases with age into adulthood [22] and we were interested in the ranging behaviour of adults only. Dispersing adults (n = 17, 8 female, 9 male) were also excluded from our analysis. Dispersal was identified if the GPS data showed that they had made a permanent move from one social group to another social group. Dispersers were identified by plotting GPS locations in ArcMap. As a dispersing badger makes exploratory forays outside their natal group’s range [55], it exhibits a temporary increase in the area over which it ranges, but this does not equate to a home range or territory. Sub-setting the dataset to exclude cubs, yearlings and dispersers resulted in a remaining dataset of 48 badgers (83 home range estimates from 48 individuals over 6974 collar-nights) for analysis. A single badger could have more than one home range estimate if it had been wearing a collar in June/July/August of multiple years. For a separate analysis of the duration of super-ranges, monthly home ranges for all badgers for all months were also estimated and plotted. Trapping records were used to estimate population density using both Minimum Number Alive (MNA) estimates [56] and Capture-Mark-Recapture (CMR) estimates using the Lincoln-Peterson method [57]. Distance between main setts was calculated using the Near Analysis tool in ArcMap. Statistical analysis was carried out in R Version 3.3.1. Home range area (km^2^) was log-transformed to normalise the data for analysis.

## Results

### General results

The study area contained badgers from 12 different social groups. Minimum Number Alive (MNA) estimates [56] give an average population density of 1.2 badgers/ km^2^. To account for individual heterogeneity in capture probabilities we also used the M(h) method [58] in the program CAPTURE [59,60], which gives an average population density of 1.8 badgers/km^2^ (Table 1). As they are based upon trapping records, these are conservative density estimates [61,62]. On average, there were slightly more females than males (0.8 males for every female). The mean distance between adjacent main setts in the study area was 1313m (range 469-2399m, SD 455m). Four badgers tested positive for TB, with one symptomatic badger being euthanized. Two asymptomatic badgers died in road traffic accidents and one remained asymptomatic to the end of the study. All had been vaccinated with BCG when first captured.

**Table 1:** Population Density Estimates (badgers/km^2^) for all badgers (adults in brackets) using the MNA [56] method and the M(h) method [58] in CAPTURE to account for individual heterogeneity.

Year	Study Area (km^2^)	MNA Population Size	MNA density (badgers/km^2^)	CAPTURE Population Size	Std. Error	95% CI	CAPTURE density (badgers/km^2^)
2010	18.52	23 (17)	1.2 (0.9)	33	3.71	30–45	1.8
2011	25.67	23 (15)	0.9 (0.6)	45	5.75	38–61	1.8
2012	22.03	30 (17)	1.4 (0.8)	40	4.23	36–55	1.8
2013	30.12	28 (22)	0.9 (0.7)	53	6.44	44–69	1.8
2014	38.25	31 (17)	0.8 (0.4)	65	6.84	56–83	1.7
2015	38.24	51 (29)	1.3 (0.8)	71	5.77	63–86	1.9
2016	32.72	50 (26)	1.5 (0.9)	53	3.71	49–65	1.6
**Mean**			**1.2** (0.7)				**1.8**
**SD**			**0.3** (0.2)				**0.09**

### Home range size

**Fig 1:**
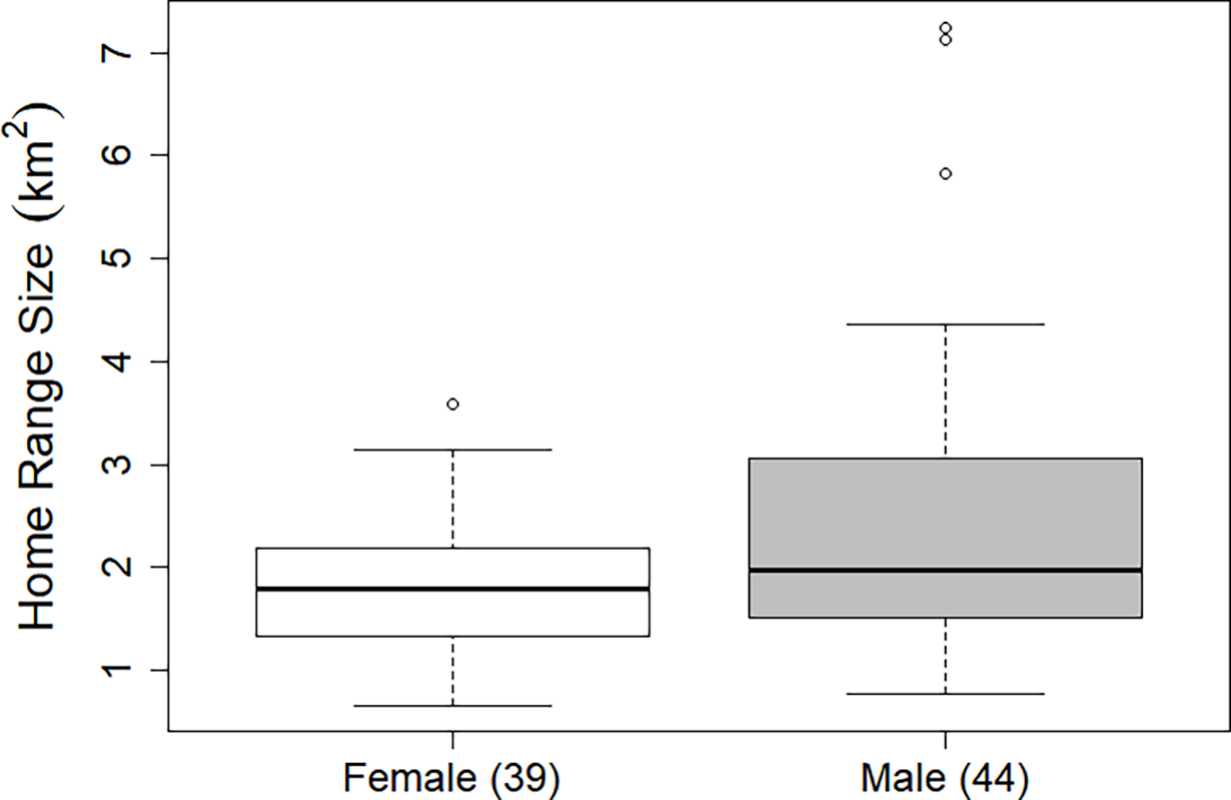
Boxplot of home range size (km^2^). Adult female badgers are represented by white boxes and adult male badgers by grey boxes. Numbers in brackets indicate sample size.

The mean number of locations used to calculate each summer home range estimate was 541.6 (range 35–2079, SD = 352.2). The mean individual summer home range size for adult badgers was 2.18km^2^ (range 0.66–7.24km^2^). The mean adult male home range and the mean adult female home range was 2.5km^2^ (range 0.78–7.23, SD = 1.48) and 1.84km^2^ (range 0.66–3.59, SD = 0.72) respectively. On average, female adult badgers ranged over a significantly smaller area than male adult badgers (t = -2.3532, df = 81, p < 0.05, Fig 1).

### Anomalies in home range size and position

**Fig 2:**
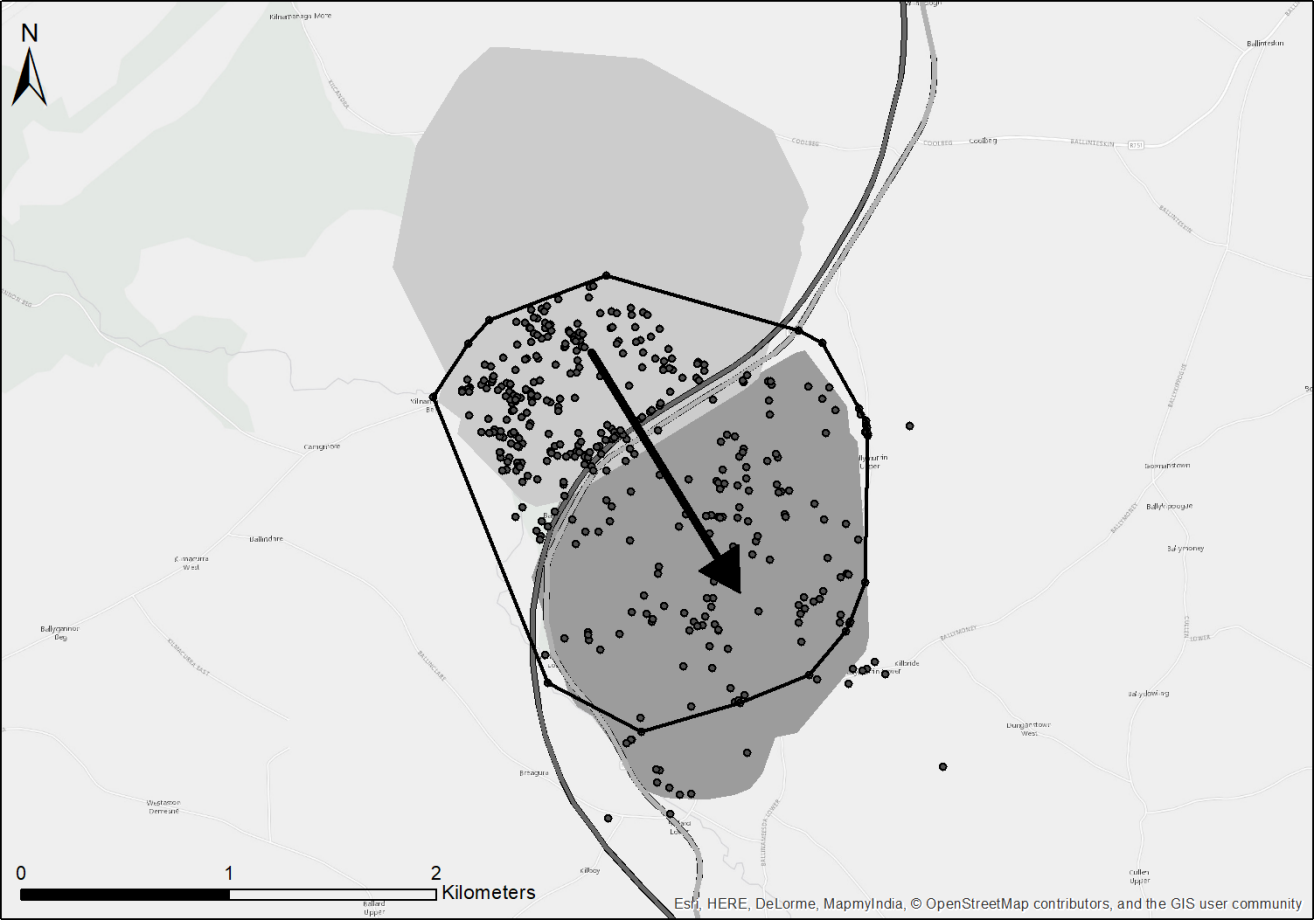
Traditional versus Super Home Ranges. Filled polygons represent traditional social group ranges, Hawthorn social group to the north and Bracken social group to the south. The dashed polygon represents the summer 2016 home range (95% MCP) of Boru, a male badger belonging to the Hawthorn social group, but who habitually ranged across the traditional boundary separating the two social groups. The dots represent Boru’s GPS locations and the arrow represents the direction of extended ranging. Thick grey lines represent the N11 and M11 roads.

**Fig 3:**
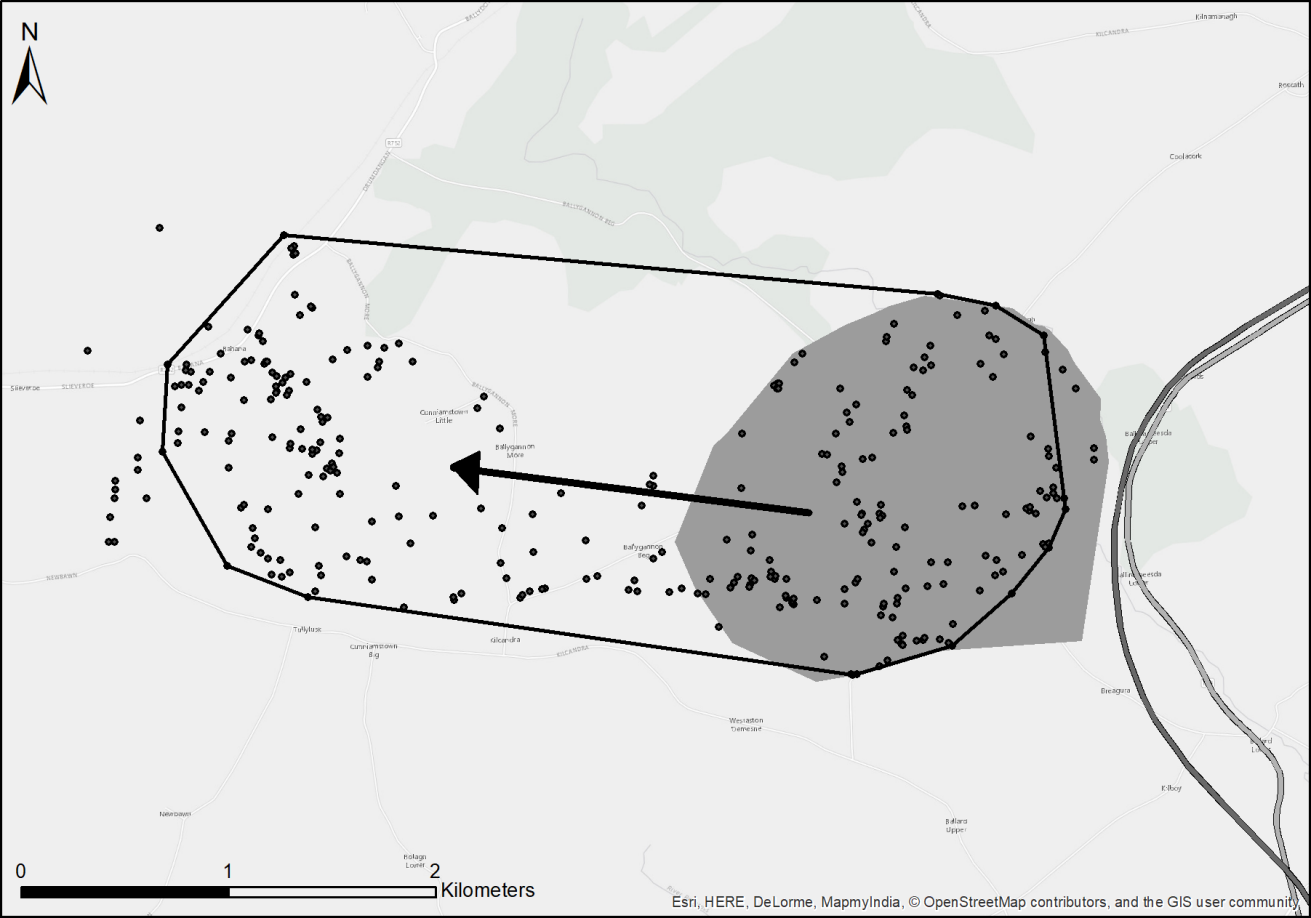
Traditional versus Super Home Ranges. The filled polygon represents the Quarry social group’s traditional range. The dashed polygon represents the summer 2013 home range (95% MCP) of Billy, a male from the Quarry social group who habitually ranged beyond the boundary of that social group. The dots represent Billy’s GPS locations and the arrow represents the direction of extended ranging. Thick grey lines represent the N11 and M11 roads.

On plotting the home ranges of adult badgers in ArcMap, two different ranging behaviours became apparent among members of the same social group. Most badgers of the same social group had home ranges that overlapped with one another (mean overlap 68%, SD = 22.5%, range = 22–100%) within the same traditional boundaries. However, some badgers showed a different behaviour, ranging across traditional social group boundaries for extended periods of time (*e*.*g*. Figs 2 and 3).

### Who are these unusual badgers?

Super-ranging occurred in all years of the study, except the first year. Of our 48 badgers, 12 different individuals, all male, habitually ranged throughout two (or more) traditional home ranges (Table 2). The ranges of these SRs crossed “traditional” social group boundaries that other members of their respective social groups rarely crossed. Further, this ranging behaviour was sustained for longer than 6 weeks. The mean age of SRs was 3 years old (range 2–5, SD = 1). The mean age of TRs *i*.*e*. adult males that did not maintain super-ranges, was 3 years old (range 2–6, SD = 1) and the mean age of adult females was 4 years old (range 2–7, SD = 1). The mean number of SRs in the study area in any given year was 2.2 (SD = 1.4) which represented on average 22% of all collared males, and 9% of all collared badgers in the study area. One individual (Roy) held two different super-ranges, both containing his natal social group’s range but extending into different social groups at different times. Plots of monthly home ranges were used to determine how long super-ranges were held. The mean length of time SRs maintained their range was 11 months (range 2–36, SD = 9.2, n = 13). The majority (75%) of SRs home ranges with alive known outcomes eventually contracted in size, two back to their original home ranges, and one to a subset of its super-range. On average it took 23 months (SD = 13) before contraction occurred. Contraction happened over the course of a few weeks. In one case it occurred overnight. The two animals with the longest held super-ranges (Billy and Ray) demonstrated three phases of ranging expansion (from traditional range to super-range), maintenance (for 2 and 3 years respectively) and subsequent contraction (from super-range to traditional range). For all other SRs, the data were incomplete (Table 2).

**Table 2:** Details of super-rangers’ (SRs) home ranges and comparison with the size of their social group-mates’ (SGMs) home ranges.

SR ID	Natal Social Group	Social Group Ranged Into	SGMs mean home ranges (km^2^)	SRs home range (km^2^)	SR Ranges relative to SGMs (%)	Duration of SR (Months)	Age*	Year(s) of Super-Ranging	Outcome
Billy	The Quarry	Outside study area	2	5.1	255	24	3	2012, 2013	Contracted
Boru	Hawthorn	Bracken	1.8	3.4	189	2	3	2016	RTA **
Brian	The Pines	Outside study area	1.6	4	250	4	2	2011	Contact lost
Dave	Bracken	Hawthorn	2	2.4	120	7	2	2013	RTA
Douglas	Hawthorn	Bracken	1.8	3.8	211	10	2	2012	RTA
Juan	The Pines	Outside & Hawthorn	1.6	7.2	450	7	2	2013	Contact lost
Leo	The Dump	Hawthorn	NA^**†**^	4.2	NA	5	Adult^‡^	2015	Contact lost
Louis	The Pines	Outside & Hawthorn	1.6	4.4	275	10	3	2014	Contracted
Michael	Ballad	Big Tree	1.4	3	214	11	2	2016	End of Study
Niall	Conifer	Big Tree	2.1	5.8	277	10	Adult^‡^	2015	Contact lost
Ray	The Briars	The Cemetery	NA^**†**^	3.4	NA	36	4	2013, 2014, 2015	Contracted
Roy	Sycamore	Driving Range	1.5	3.6	240	9	3	2011	Contact lost
Roy	Sycamore	The Cemetery	NA^**†**^	1.9	NA	7	5	2013	RTA

* Where more than one year of data, age in first year of super-range.

**Road traffic accident

^†^ No collared adult, non-dispersal social group-mates for comparison.

^‡^ Estimated to 3 years of age or older, but no finer estimate made.

Some groups appeared to be more likely than others to produce SRs than other groups. Of the twelves individuals that engaged in super-ranging, three belonged to The Pines social group and two to the Hawthorn social group. However, we can see no obvious ecological explanation for this.

### Home range size in the context of SRs

**Fig 4:**
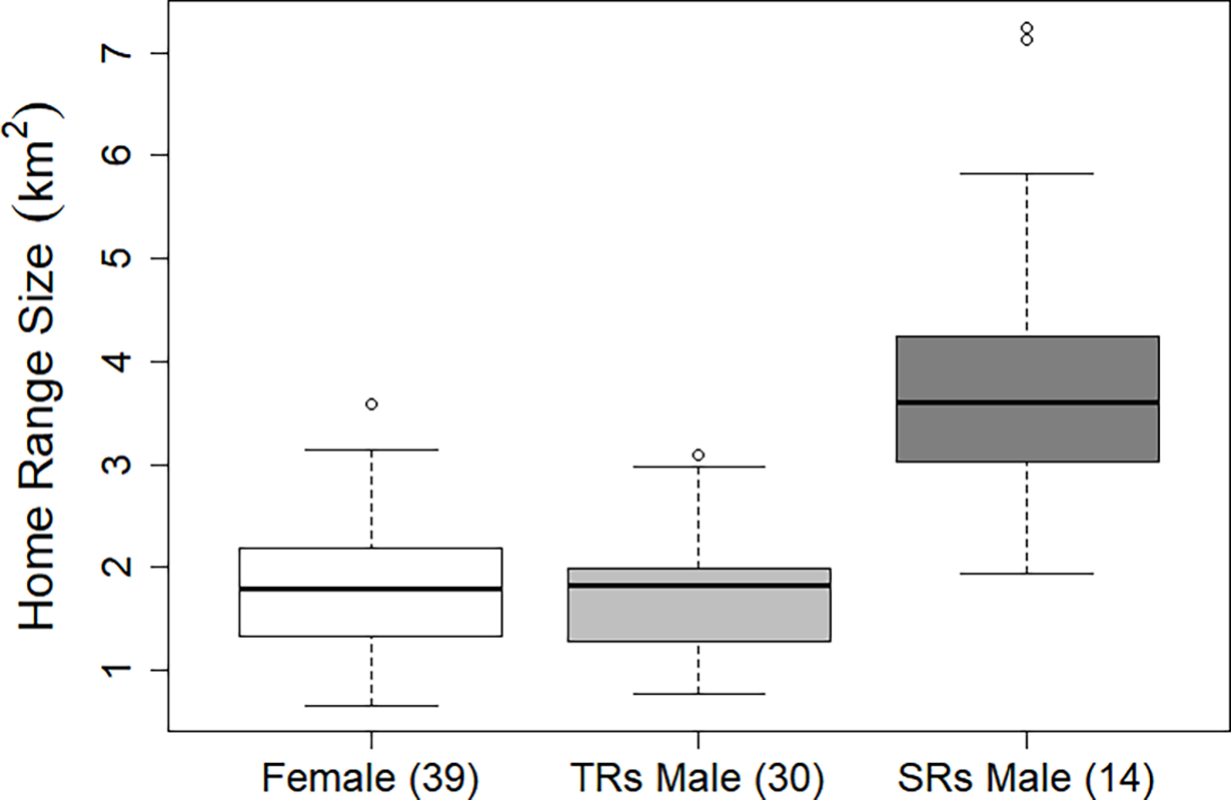
Boxplot of Home Range Size (km^2^). Here, female badgers are represented by white, TRs by light grey and SRs by dark grey boxes. Numbers in brackets indicate sample size.

Home range sizes were re-analysed in the context of two male ranging strategies, super-ranging and traditional ranging. The home range sizes of SRs were significantly larger than other badgers (ANOVA: (F(2, 80) = 25.21, p < 0.001, Fig 4). SRs maintained a mean home range of 3.95 km^2^ (range 1.94–7.23, SD = 1.62) while TRs maintained a mean home range of 1.74km^2^ (range 0.78–3.09, SD = 0.58). Adult females maintained a mean home range of 1.84km^2^ (range 0.66–3.59, SD = 0.72). Both adult female badgers and TRs ranged over significantly smaller areas than SRs (Tukey post hoc, p = 0.00 Fig 4), but there was no significant difference in home range size between females and TRs (Tukey post hoc, p = 0.959, Fig 4).

## Discussion

The ranging behaviour of badgers is of direct importance to the both intra- and interspecific transmission of TB. Here we present evidence for a previously unrecognised ranging strategy in badgers. While initial results suggested there was a sex difference in ranging behaviour with females ranging over smaller areas than males, it became clear that, in our study population, male badgers were not all ranging in the same way. Most males (TRs) would range within traditional social group boundaries, maintaining an individual home range like that of current and historical members of their social group. However, on average 22% of males (SRs) consistently ranged far beyond these boundaries, using more than one social group’s territory at the same time. Multiple types of movement have been defined for badgers [39,40,63–66] including movement between social groups. In badgers, movement between groups can occur for several reasons. Foraging, extra-territorial mating and exploration in preparation for dispersal all take individuals outside their traditional group boundary for short periods of time [55,64,67,68]. Foraging forays are very short-lived; badgers tend to return to a rich-food patch each night for a few nights until it has been depleted. While mating can occur at any time between blastocyst implantation and parturition [69], mating forays tend to peak in Spring when females upon giving birth come back into season, and again in late summer/early autumn, coinciding with another peak in mating [70,71]. Although our data contains examples of male badgers visiting neighbouring setts in February, these extra-territorial excursions for reasons of mating are again only of 24 hours duration at most. Finally, dispersal can cause badgers to travel outside of their territory. In our study population, 14.7% of badgers dispersed over the course of the study period, permanently moving from one social group to another [55,72]. Of the badgers that were wearing GPS collars for the dispersal event itself, the time taken to move ranged between one night to six weeks. If dispersal was not instantaneous, the process was characterised by the badger making exploratory forays into one or several different social groups, usually lasting between 1–3 days each time, before finally settling in their new group.

None of these patterns of ranging behaviour are consistent with that of SRs, which we describe here. While it is possible that these badgers were engaged in a greatly extended dispersal process and acting as “floaters”, a strategy seen in wolves [73,74], we do not believe this to a good description of our super-rangers behaviour. In our study area, 90% of male dispersers moved to an adjacent social group, and ultimately no longer ranged within the bounds of their original social group. Super-ranges were maintained for considerably longer than even the increases in home range caused by the exploratory phase of dispersal, sometimes for several years, and they did not culminate in badgers changing social group. SRs were regularly trapped at main setts within the ranges of multiple social groups, showing that they were maintaining a presence in each of the social groups within their super-range. The SRs’ ranging was also not consistent with extra-territorial feeding at ephemeral food patches or with mating forays as the badgers patrolled the full extent of their expanded range consistently over several months. Differences in ranging behaviour in badgers have been related to TB status, with ranging behaviour becoming increasingly abnormal as the disease progresses [75,76]. Garnett et al.[76] found that infected badgers (n = 8) maintained home ranges 50% larger than uninfected badgers (n = 8), and these ranges extended significantly into neighbouring territories. However, only one of our 12 super-rangers tested positive for TB while remaining asymptomatic, so their ranging behaviour cannot be explained by a positive disease status or by progressing disease. Neither was the behaviour an artefact of trapping, as no collared badgers altered their ranging behaviour in response to being trapped nor to wearing GPS collars [77]. It is apparent that badgers using a super-ranging strategy were using more than their own social group’s range, maintaining a consistent presence in an area covering space used by multiple discrete social groups.

We have considered several possible motivations for super-ranging. SRs may be subordinate males that are being partially excluded from their natal range by a dominant male and are effectively “floaters” [73]. However, rather than dispersing completely, they maintain contact with their original social group due to philopatry [65]. Another possibility is that SRs are dominant males that are actively defending extremely large territories over large periods of time. It is possible that SRs filled a vacuum left by the loss of the dominant male in the neighbouring territory, perhaps allowing them access to a greater number of females [22,37,40,78]. In five cases, we know that the resident male died or disappeared before the SR expanded into his range. In three cases however, the SR moved into neighbouring ranges where resident males were present, although in all cases they were cubs or yearlings. In one case, an SR contracted his range upon the appearance of another SR. Erlinge & Sandell [79], describe in another mustelid, the stoat, differences in male social organisation that is evident during the females’ discrete receptive period. Males are either dominant “roamers” who gain access to many females, or “stayers” who maintain contact with just a few females. As badgers can mate at any time of the year, it is possible that super-rangers represent an extended version of “roamers”, but which is maintained all year. This would result in super-ranging being one of two evolutionarily stable mating tactics that coexists within the same population [80]. In our study, we cannot distinguish between these motivations. The nature of GPS data can only give us positional information, and does not show whether a badger has been marking at latrines, fighting or peacefully co-existing with conspecifics. However, it is notable that the two males with the longest held super-ranges accumulated many scars from bite-wounding. Despite these wounds, they were both very healthy and maintained their super-ranges for long periods of time (24 and 36 months respectively). We tentatively suggest, therefore, that these males were in fact defending their large territories. However, further behavioural data (*e*.*g*. camera trapping of marked individuals at setts and latrines and the use of proximity collars) is required to demonstrate which explanation is correct.

### Evidence for SRs in other studies?

A review of the ranging literature revealed a number of unusual ranging behaviours which might be explained by super-ranging. The majority of these studies were based on radio-tracking or trapping data which, by their nature, give only short-term or snapshot views of ranging behaviour [66] Cheeseman et al. [39], identified a category of movement in UK badgers “between non-associated, adjacent social groups at the same time”, estimating that approximately 9.3% of recaptured males engaged in this type of behaviour. They also found one female in the rural population engaging in this type of ranging behaviour (0.06% of that population). Rogers et al.’s [40] investigation of movement in a high-density UK population using capture data, defined badgers that moved between social groups other than their own as “frequent movers”, constituting 4.8% of all badgers that moved. Other studies have also noted individuals of ambiguous social group membership based on trapping records and/or ranging behaviour. Kruuk [25] noted two males that appeared to belong to two different social groups at the same time, spending their day in either of the main setts, with ranges that completely incorporated those of the females from both social groups. Davison et al. [34] described a male badger in urban Brighton that could not be assigned to a single social group as it was using the main setts and above ground home ranges of two groups. In Luxembourg, Frantz et al. [42] noted that an earlier study of their population [81] reported one male that had a range that encompassed two of the social groups in their later study. They suggested group fission and a flexible social system as an explanation. In Spain, Revilla & Palomares [21,78] noted that males would expand their range into adjacent ranges, given the opportunity *e*.*g*. if a neighbouring male died. In Ireland, Elliot et al. [38] describe overlapping home ranges of two badgers from different social groups, a male and a female, the male having a home range that was nearly an order of magnitude larger than the female’s home range (334ha v 39ha). They proposed that their data were indicative of social fluidity in badgers. It is possible that these all may in fact have been examples of SR-type ranging behaviour, which without the benefit of long-term monitoring remained obscure. However, we also cannot discount the possibility that at least some of these may have been badgers recorded in the process of dispersal.

Recent studies have been employing more advanced technology. A GPS tracking study [77], noted one individual that could not be assigned to a social group based on trapping records and collar data. Unfortunately, the ranging behaviour of this individual is not described and it is unknown whether this was a dispersing or super-ranging individual. A study [82] using RFID contact collars found that at high density, connectivity between social groups may be much greater and territoriality much less than conventionally suggested [83]. Their network analysis showed evidence of trans-border “super-groups” *i*.*e*. badgers from these groups frequently transgressed territorial boundaries [82,84]. However, 20–48% of collared badges were recorded at main setts other than their own, rather than a few highly connected individuals [82]. The fact that there appeared to be a connection between The Pines and Hawthorn and between Hawthorn and Bracken social groups may appear consistent with idea of clustering of super-groups. However, in this instance it would be due to the ranging of a few individuals, the super-rangers, rather than a large proportion of those social groups. As we have no reason to suggest that our badgers behave differently from others living at a similar density, we would expect to find SRs in other study populations. Unfortunately, until long-term GPS tracking of individual badgers is carried out, evidence for the more widespread occurrence of this trait in other populations will remain lacking.

### Implications for transmission and control of TB

Although it remains unclear why some male badgers maintain super-ranges while others do not, the implications of such ranging behaviour may be significant for the transmission and control of TB in badgers. Asymmetries in the contact structure within a population affect the way in which diseases are transmitted through a social network [85]. The organisation of badgers into territorial social groups, at least at higher densities, appears to limit the spread of TB because it lowers disease transmission rates between groups [32,86,87]. However, badger movements into and out of neighbouring social groups is associated with increased prevalence of TB in these groups [40,66]. The implication is that movement between social groups increases direct contact between individuals from different social groups, therefore increasing the rate of disease transmission.

In non-dispersing badgers, home ranges normally map directly onto territory boundaries [24,88]. Social group members mark and patrol these boundaries and may actively exclude conspecifics through fighting [25,83] though the degree to which territoriality is expressed may be a function of population density [31,89]. Given that bite-wounding is the second most frequent mode of TB transmission among badgers [6] and that movement between social groups has been associated with increased TB prevalence [40,66], SRs ranging throughout more than one social group’s range can expect to have a larger border to defend and higher encounter rates with individuals from different social-groups than TRs. If these interactions are agonistic, the risk of disease transmission is increased. Further, these males have to expend a lot of energy maintaining ranges that are significantly larger than TR males and adult females. The stress associated with maintaining these ranges may impact the degree to which the disease manifests itself, as a stressed infected badger is more likely to be shedding *M*. *bovis* bacilli than unstressed individuals [66,90]. Super-rangers may therefore act as super-spreaders of TB infection. Our data suggest that maintaining a super-range is unsustainable in the long-term, with SRs’ home ranges eventually contracting towards their original size. Indeed, the two badgers with the longest records for super-ranges both consistently bore fight-wounds when trapped and their ranges eventually contracted. This contrasts with the lack of bite-wounding in super-groups as described by Ellwood et al. [82].

## Conclusion

In addition to the implications for the spread of TB, our findings increase the knowledge of badger ranging behaviour by highlighting a hitherto unrecognised male ranging strategy. It is possible that apparent sex differences in territory size found in some studies are due to the presence of SR males in the population. While it may be difficult to identify SRs retrospectively without the use of GPS collars, we recommend that other researchers using such collars should look for the presence of SRs in their study populations. The implications of the presence of two distinct ranging strategies by different males, and the consequently improved understanding of badger sociality and ecology, are likely to be profound. In addition, the influence of SRs in carrying infection between social groups would need to be incorporated into epidemiological modelling, and the formulation of disease control policy.

## Supporting information

S1 Table Seasonal Home Range Sizes (km^2^) of badgers in the study area. (CSV)

S2 Table Summer Home Range Size (km^2^) dataset for N11 badgers. (CSV)
